# Tendência temporal da avaliação do manejo adequado para diagnóstico e tratamento da tuberculose na atenção primária à saúde no Brasil entre 2012-2018 

**DOI:** 10.1590/0102-311XPT087723

**Published:** 2024-03-11

**Authors:** Larissa Picanço, Rinelly Pazinato Dutra, Mirelle de Oliveira Saes

**Affiliations:** 1 Universidade Federal do Rio Grande, Rio Grande, Brasil.; 2 Hospital Escola, Universidade Federal de Pelotas, Pelotas, Brasil.

**Keywords:** Tuberculose, Atenção Primária à Saúde, Qualidade, Acesso e Avaliação da Assistência à Saúde, Tuberculosis, Primary Health Care, Health Care Quality, Access and Evaluation, Tuberculosis, Atención Primaria de Salud, Calidad, Acceso y Evaluación de la Atención de Salud

## Abstract

O objetivo do estudo foi analisar a presença de infraestrutura e processo de trabalho adequados na atenção primária à saúde (APS) para o diagnóstico, o monitoramento e o tratamento da tuberculose (TB) no Brasil de 2012 a 2018. Estudo de tendência temporal realizado com dados das unidades básicas de saúde (UBS) avaliadas nos ciclos I (2012), II (2014) e III (2018) do Programa Nacional de Melhoria do Acesso e da Qualidade da Atenção Básica (PMAQ-AB). Foi empregada a regressão de mínimos quadrados ponderada por variância para estimar as mudanças anuais, em pontos percentuais da infraestrutura e processo de trabalho adequado da TB em relação à macrorregião, ao porte do município e ao Índice Municipal de Desenvolvimento Humano e cobertura da Estratégia Saúde da Família. A amostra foi composta por 13.842 UBS e 17.202 equipes de saúde no ciclo I, 24.055 UBS e 29.778 equipes no II e 28.939 UBS e 37.350 equipes no III. Observou-se melhora gradual na proporção de infraestrutura e processo de trabalho ao atendimento da TB ao longo dos três ciclos do PMAQ-AB; contudo, nenhum local está integralmente adequado. A maior tendência de infraestrutura adequada foi verificada na Região Sul e no ano de 2018, em que 76,5% das UBS tinham todos os instrumentos para o cuidado à TB. A maior tendência de processo de trabalho adequado foi na Região Norte e no ano de 2018, em que 50,8% das equipes tinham a totalidade de itens para o cuidado à TB. O Programa Nacional de Controle da Tuberculose e o PMAQ-AB contribuíram para tais avanços, mas ainda é necessário o fomento de políticas públicas que garantam a melhoria contínua da assistência à TB na APS e a eficácia das medidas de controle e prevenção da doença.

## Abstract (en)

This study aimed to analyze the presence of infrastructure and adequate work processes in primary health care (PHC) for the diagnosis, monitoring, and treatment of tuberculosis (TB) in Brazil from 2012 to 2018. This is a temporal trend study carried out with data from basic health units (BHU) evaluated in the cycles I (2012), II (2014), and III (2018) of the Brazilian National Program for Improvement of Access and Quality of Basic Care (PMAQ-AB). Variance-weighted least-squares regression was used to estimate annual changes, in percentage points, of the infrastructure and adequate work process of TB in relation to the macroregion, municipality size, Municipal Human Development Index, and Family Health Strategy coverage. The sample consisted of 13,842 BHU and 17,202 health teams in cycle I; 24,055 BHU and 29,778 teams in cycle II; and 28,939 BHU and 37,350 teams in cycle III. There was a gradual improvement in the proportion of infrastructure and work process for TB care over the three cycles of the PMAQ-AB, but none of the sites is fully adequate. The greatest trend of adequate infrastructure was observed in the South Region, and in 2018, 76.5% of the UBS had all the instruments for TB care. The greatest trend of adequate work process was in the North Region, and in 2018, 50.8% of the teams had all the items for TB care. The Brazilian National Program for Tuberculosis Control and the PMAQ-AB have contributed to these advances, but there is still a need to promote public policies that ensure the continuous improvement of TB care in PHC, the effectiveness of TB control and prevention measures.

## Resumen

El objetivo de este estudio fue analizar la presencia de infraestructura y proceso de trabajo adecuado en la atención primaria de salud (APS) para el diagnóstico, monitoreo y tratamiento de la tuberculosis (TB) en Brasil entre los años 2012 y 2018. Estudio de tendencia temporal realizado con datos de las unidades básicas de salud (UBS), evaluadas en los ciclos I (2012), II (2014) y III (2018) del Programa Nacional de Mejoría de Acceso y Calidad de la Atención Básica (PMAQ-AB). Se utilizó la regresión de mínimos cuadrados ponderada por varianza para estimar los cambios anuales, en puntos porcentuales de la infraestructura y el proceso de trabajo adecuado de la TB en relación con la macrorregión, el tamaño del municipio, el Índice Municipal de Desarrollo Humano y la cobertura de la Estrategia de Salud de la Familia. La muestra se compuso de 13.842 UBS y 17.202 equipos de salud en el ciclo I, 24.055 UBS y 29.778 equipos en el ciclo II y 28.939 UBS y 37.350 equipos en el ciclo III. Se observó una mejoría gradual en la proporción de infraestructura y proceso de trabajo en la atención de la TB a lo largo de los tres ciclos del PMAQ-AB, sin embargo, ningún local está completamente adecuado. Se verificó la mayor tendencia de infraestructura adecuada en la Región Sur y, en 2018, el 76,5% de las UBS tenían todas las herramientas para el cuidado de la TB. La Región Norte tuvo la mayor tendencia de proceso de trabajo adecuado y, en 2018, el 50,8% de los equipos tenían todo lo necesario para el cuidado de la TB. El Programa Nacional de Control de la Tuberculosis y el PMQA-AB contribuyeron para estos avances, pero aún es necesario promover políticas públicas que aseguren la mejoría continua de la asistencia de la TB en la APS y la eficacia de las medidas de control y prevención de la enfermedad.

## Introdução

A tuberculose (TB) é uma doença infectocontagiosa, considerada como um problema de saúde pública global ^1^^,^^2^. Embora o percentual de sucesso de tratamento com esquema medicamentoso seja de 85%, é preocupante o aumento dos casos novos e da mortalidade da doença ^2^^,^^3^. Estima-se que, mundialmente, 10,6 milhões de pessoas desenvolveram TB no ano de 2021, um aumento de 4,5% em relação a 2020. Além disso, o número de mortes por TB aumentou entre 2019 e 2021, o que pode estar atrelado ao contexto da pandemia da COVID-19, uma vez que a emergência sanitária prejudicou o acesso ao diagnóstico e ao tratamento da TB em todo o mundo ^2^.

O Brasil é um dos 30 países com alta carga de TB e coinfecção com o HIV ^4^. Ações governamentais têm sido desenvolvidas desde o ano de 1996, como o Plano Emergencial para o Controle da Tuberculose, e, em 1999, ocorreu o lançamento do Programa Nacional de Controle da Tuberculose (PNCT), que tem como objetivo estabelecer diretrizes e tratamento padronizados para o controle da doença ^5^. Além disso, no ano de 2021, o Ministério da Saúde atualizou o Plano Nacional pelo Fim da Tuberculose, estabelecendo estratégias nacionais e metas para o período de 2021 a 2025, alinhadas à Estratégia Global pelo Fim da TB e de acordo com o cenário socioeconômico brasileiro ^5^.

Há de se destacar, contudo, que a aplicabilidade das metas a serem cumpridas é um desafio perante o aumento da proporção de indivíduos em situação de pobreza e extrema pobreza no Brasil, segundo síntese dos indicadores sociais do Instituto Brasileiro de Geografia e Estatística (IBGE) ^6^. As evidências mostram que as pessoas que vivem em situação de pobreza apresentam maior possibilidade de contrair a doença ^7^^,^^8^. Nessa perspectiva, tal cenário é preocupante, uma vez que o Brasil é demarcado por históricas e persistentes desigualdades ^9^, e a TB está intimamente ligada aos determinantes sociais de saúde ^7^.

Somado às condições socioeconômicas, o alcance das metas propostas para o combate à TB enfrenta desafios relacionados à capacidade de diagnóstico e manutenção do tratamento ^5^. A análise da série histórica dos desfechos de TB multidroga resistente e resistente à rifampicina, de 2010 a 2019, no Brasil, demonstra preocupação, uma vez que o percentual de tratamento completo teve uma queda de 16,3% e o abandono ao tratamento aumentou 7,8% ^10^. No cenário mundial, o aumento da carga de TB resistente a medicamentos entre 2020 e 2021 é alarmante, com aproximadamente 450 mil novos casos ^2^ e uma estimativa de prevalência global combinada de 11,6% ^11^.

A TB é uma doença de notificação obrigatória no país e o perfil da infecção está relacionado com a capacidade dos serviços de saúde de detectar e notificar os casos ^12^. Nessa perspectiva, o Ministério da Saúde recomenda a descentralização das ações de detecção, diagnóstico e acompanhamento da TB para a atenção primária à saúde (APS), visando ampliar a abrangência e a efetividade dessas ações ^5^. Entretanto, a oferta de atenção à doença ainda é realizada de maneira fragmentada, reativa e episódica ^12^ e os coeficientes de incidência da TB apresentam uma importante heterogeneidade entre os estados ^10^. No ano de 2022, os maiores coeficientes de incidência de TB (casos por 100 mil habitantes) foram observados no Amazonas (84,1), em Roraima (75,9) e no Rio de Janeiro (68,6) ^10^.

Embora existam diretrizes e metas estabelecidas para o controle efetivo da TB ^2^^,^^5^^,^^10^, é evidente a escassez de estudos que avaliem de forma abrangente a implementação e aplicabilidade dessas políticas na APS ^12^^,^^13^. Essa importante lacuna na literatura limita a avaliação adequada dos serviços de saúde, a compreensão dos desafios enfrentados e a identificação de áreas com necessidades prioritárias para o desenvolvimento de estratégias mais eficazes. A análise desses aspectos pode fornecer informações valiosas para o aprimoramento do manejo adequado da TB na APS.

Portanto, este estudo tem como objetivo analisar a presença de infraestrutura e processo de trabalho adequado na APS para o diagnóstico, monitoramento e tratamento da TB no Brasil entre os anos de 2012 e 2018.

## Método

Trata-se de um estudo de tendência temporal com dados da avaliação externa dos três ciclos do Programa Nacional de Melhoria do Acesso e da Qualidade da Atenção Básica (PMAQ-AB). Esse programa foi instituído pela *Portaria GM/MS nº 1.654*^14^, de 19 de julho de 2011, com o objetivo de estimular a expansão do acesso e promover a melhoria da qualidade da APS. O ciclo I do programa ocorreu de maio a dezembro de 2012; o ciclo II, de novembro de 2013 a outubro de 2014; e o ciclo III, de julho 2017 a agosto de 2018. Os municípios eram convidados a participar voluntariamente. A adesão dos municípios brasileiros ao PMAQ-AB foi crescente, sendo de 71% no ciclo I, 91% no ciclo II e 96% no ciclo III.

Os dados da avaliação externa foram coletados por 41 instituições de Ensino Superior brasileiras, sendo essas coletas lideradas por oito delas ao longo dos três ciclos - Universidade Federal de Pelotas (UFPel), Universidade Federal do Rio Grande do Sul (UFRGS), Fundação Oswaldo Cruz (Fiocruz), Universidade Federal de Minas Gerais (UFMG), Universidade Federal da Bahia (UFBA), Universidade Federal do Rio Grande do Norte (UFRN), Universidade Federal de Sergipe (UFS) e Universidade Federal do Piauí (UFPI).

O instrumento de pesquisa foi desenvolvido pelo Ministério da Saúde em parceria com as instituições federais líderes do estudo. Os questionários eram enviados automaticamente para o servidor central do Ministério da Saúde, sendo monitorados continuamente para assegurar a qualidade e a consistência por meio de um validador eletrônico. A coleta de dados foi realizada a partir de um questionário eletrônico, aplicado por entrevistadores devidamente capacitados. Os questionários para os três ciclos continham perguntas específicas sobre a gestão municipal, unidade básica de saúde (UBS), equipe de trabalho e usuários, e abrangiam temáticas prioritárias da Política Nacional de Atenção Básica (PNAB). Tais informações foram divididas em três módulos: o primeiro sobre a infraestrutura da UBS (aplicado ao responsável da UBS); o segundo sobre o processo de trabalho da equipe (aplicado ao responsável da equipe de saúde); e o terceiro, que avaliou a qualidade do cuidado recebido por meio da entrevista com os usuários do serviço.

Especificamente para a elaboração deste estudo, foram utilizadas informações sobre o cuidado à TB dos três ciclos. Foram coletadas informações sobre a infraestrutura da UBS (módulo 1) e sobre o processo de trabalho das equipes (módulo 2). As variáveis foram selecionadas de modo a construir dois desfechos sintéticos que fossem capazes de avaliar a infraestrutura da unidade e o processo de trabalho das equipes com relação ao diagnóstico, à gestão e ao tratamento do usuário com TB. Sendo eles:

(1) Infraestrutura adequada para diagnóstico, manejo e tratamento de pacientes com TB. Definido a partir da resposta afirmativa à presença de estetoscópio; equipamentos de proteção individual (EPI), sendo eles: máscaras descartáveis, luvas, óculos de proteção e avental; e teste rápido de HIV. As três variáveis foram avaliadas separadamente e, de forma conjunta, formaram o escore de infraestrutura da TB, sendo considerada como tendo infraestrutura adequada a resposta afirmativa à presença dos itens acima investigados em cada ciclo. Ressalta-se que a variável EPI não foi coletada no ciclo II e, portanto, não pode ser indicada como infraestrutura inadequada.

(2) Processo de trabalho adequado dos profissionais e da equipe para manejo e tratamento dos pacientes com TB. Definido a partir da resposta afirmativa aos seguintes itens: presença do exame de baciloscopia, radiografia, sorologia para HIV; estabelecimento dos critérios de risco e vulnerabilidade para a definição da quantidade de pessoas sob a responsabilidade da equipe (territorialização); realização de grupos com o enfoque de orientar sobre as doenças transmissíveis (incluindo TB); busca ativa de usuários com sintomas respiratórios; coleta da primeira amostra de escarro para diagnóstico da TB na UBS; registro do número de usuários com TB; ficha de notificação de diagnóstico da TB; realização de tratamento diretamente observado (TDO); busca ativa faltosos do TDO. Para o desenvolvimento do desfecho sintético, foi considerado como processo de trabalho adequado a resposta afirmativa à presença dos itens acima investigados em cada ciclo. Cabe destacar que a variável coleta da primeira amostra de escarro para diagnóstico da TB na UBS não foi coletada no ciclo I; as variáveis EPI e busca ativa de usuários com sintomas respiratórios não foram coletados no ciclo II e a variável realização de grupos com enfoque de orientar sobre doenças transmissíveis (incluindo TB) não foi coletada no ciclo III.

A prevalência do desfecho sintético da infraestrutura foi calculada de acordo com o número de unidades avaliadas, e o desfecho processo de trabalho adequado, em relação ao número de equipes de saúde investigadas. Foram analisadas como exposições, as macrorregiões geográficas (Norte, Nordeste, Centro-oeste, Sudeste e Sul); o tamanho do município (até 10.000 habitantes; de 10.001 a 30.000; de 30.001 a 100.000; de 100.001 a 300.000; e mais de 300.000 habitantes); o Índice de Desenvolvimento Humano Municipal - IDH-M (baixo: até 0,554; médio: de 0,555 a 0,699; alto: de 0,700 a 0,799; e muito alto: 0,800 ou mais); e cobertura pela Estratégia Saúde da Família (ESF) (até 50%; 50,1% a 75%; 75,1% a 99,9%; 100%).

Para a análise dos dados, inicialmente foram descritas as variáveis que compuseram cada um dos desfechos sintéticos nos três ciclos do PMAQ-AB e, após, calculou-se a prevalência de cada desfecho sintético em cada ciclo de avaliação. Para identificar a diferença entre os ciclos, foi feita a análise de tendência a partir da regressão mínima quadrada ponderada, para estimar as variações anuais percentuais nos valores de prevalência. Como significância estatística foram adotados os valores de p < 0,05. Todas as análises foram realizadas utilizando-se o pacote estatístico Stata, versão 16.0 (https://www.stata.com).

Os três ciclos do PMAQ-AB foram aprovados por Comitês de Ética em Pesquisa (CEP). O ciclo I foi aprovado pelo CEP da Faculdade de Medicina da UFPel (ofício oficial nº 38/2012); o II teve parecer favorável emitido pelo CEP da Universidade Federal de Goiás (parecer nº 487.055) em 12 de dezembro de 2013; e o ciclo III foi aprovado pelo CEP da Faculdade de Medicina da UFPel (parecer nº 2.453.320). Todos os participantes assinaram um termo de consentimento livre e esclarecido.

## Resultados

A amostra estudada foi composta por 13.842 UBS e 17.202 equipes de saúde da família no ciclo I (2012), 24.055 UBS e 29.778 equipes no ciclo II (2014), e 28.939 UBS e 37.350 equipes no ciclo III do PMAQ-AB. A Tabela 1 apresenta a distribuição das UBS e equipes de acordo com a região, porte populacional do município, IDH-M e cobertura de ESF em cada um dos três ciclos de avaliação.

**Tabela 1:** Distribuição das unidades básicas de saúde (UBS) e das equipes avaliadas de acordo com as variáveis independentes. Avaliação externa do Programa Nacional de Melhoria do Acesso e da Qualidade da Atenção Básica (PMAQ-AB), Brasil, 2012, 2014 e 2018.

Características	Ciclo I (2012)		Ciclo II (2014)		Ciclo III (2018)	
	UBS	Equipes	UBS	Equipes	UBS	Equipes
	%	%	%	%	%	%
Região [n]	[13.842]	[17.202]	[24.055]	[29.778]	[28.939]	[37.350]
Norte	5,8	4,3	7,0	6,0	7,8	6,9
Nordeste	36,8	32,3	40,3	36,2	41,7	37,1
Sudeste	33,4	38,2	30,0	33,9	28,9	33,1
Sul	17,3	17,0	15,0	15,1	14,3	14,6
Centro-oeste	6,8	8,2	7,6	8,7	8,5	8,3
Tamanho da população (habitantes) [n]	[13.842]	[16.993]	[24.051]	[29.774]	[28.856]	[37.257]
Até 10.000	17,1	15,0	14,8	13,3	14,4	12,7
De 10.001 a 30.000	32,6	27,8	32,6	27,8	32,2	27,0
De 30.001 a 100.000	24,0	20,7	26,0	22,9	27,0	23,8
De 100.001 a 300.000	11,9	12,1	12,8	13,3	12,8	14,0
Mais de 300.000	14,5	24,6	13,8	22,7	13,6	22,7
IDH-M [n]	[13.842]	[16.949]	[24.054]	[29.777]	[28.848]	[37.249]
Baixo (até 0,554)	2,2	1,9	5,7	4,9	6,3	5,4
Médio (de 0,555 a 0,699)	37,4	32,0	50,3	43,9	51,3	44,8
Alto (de 0,700 a 0,799)	39,6	37,2	38,8	41,0	37,8	41,1
Muito alto (0,800 ou mais)	20,8	29,0	5,2	10,2	4,6	8,9
Cobertura de ESF (%) [n]	[13.842]	[17.202]	[24.055]	[29.777]	[28.856]	[37.257]
Até 50	22,9	28,5	15,6	29,8	10,9	15,9
De 50,1 a 75	24,2	25,3	17,5	23,7	17,0	19,7
De 75,1 a 99,9	22,1	19,4	20,5	19,4	21,3	21,1
100	30,8	26,8	46,4	27,1	50,9	44,2

ESF: Estratégia Saúde da Família; IDH-M: Índice de Desenvolvimento Humano Municipal.

Nos três ciclos avaliados, houve maior concentração das UBS na Região Nordeste (36,8%, 40,3% e 41,7%, respectivamente). Já em relação às equipes, no ciclo I, foram mais concentradas na Região Sudeste (38,2%) e, nos ciclos II e III, na Região Nordeste, (36,2% e 37,1% respectivamente). Em relação ao porte, cerca de 32% das UBS e 27% das equipes estavam localizadas em municípios com população entre 10.001 e 30.000 habitantes em todos os ciclos avaliados. Aproximadamente 77% das UBS concentravam-se em localidades com IDH-M entre médio e alto, no ano de 2013, e por volta de 89,1%, em 2018. As equipes de saúde distribuíram-se de forma similar: 69,2% estavam localizadas em municípios com IDH-M médio e alto, em 2013, e 85,9%, em 2018. Por fim, observa-se que as UBS se concentraram principalmente em territórios com 100% de cobertura de ESF em todos os três ciclos do PMAQ-AB (Tabela 1).

Em relação à prevalência de infraestrutura adequada para diagnóstico, tratamento e monitoramento da TB, é possível observar que praticamente todas as UBS dispunham de estetoscópio em todos os ciclos avaliados. Destaca-se que pelo menos um quarto das unidades não apresentam EPI para o cuidado de pacientes com TB, tanto no ciclo I quanto no ciclo III. Por sua vez, houve aumento de quase 60p.p. na disponibilidade de testes rápidos de HIV entre o ciclo I e o ciclo III. Em relação à infraestrutura adequada, verificou-se um aumento progressivo no percentual em cada ciclo avaliado, partindo de 12,1% no I, para 29,4% no II, e chegando a 54,6% no ciclo III (Tabela 2).

**Tabela 2:** Prevalência e razões de prevalência para adequação da infraestrutura das unidades básicas de saúde (UBS) e do processo de trabalho das equipes para diagnóstico e tratamento da tuberculose. Avaliação externa do Programa Nacional de Melhoria do Acesso e da Qualidade da Atenção Básica (PMAQ-AB), Brasil, 2012, 2014 e 2018.

Variável	Ciclo I (2012)		Ciclo II (2014)		Ciclo III (2018)	
	%	IC95%	%	IC95%	%	IC95%
Estetoscópio	98,2	98,0-98,4	98,6	98,4-98,7	99,5	99,4-99,5
EPI	71,9	71,1-72,6	-	-	69,1	68,5-69,7
Teste rápido de HIV	14,7	14,1-15,3	30,2	29,6-30,8	73,2	72,7-73,7
Infraestrutura adequada	12,1	11,6-12,6	29,4	29,3-30,4	54,6	54,0-55,2
Baciloscopia	96,7	97,4-97,9	96,8	96,6-97,0	98,2	98,1-98,3
Raio-X	95,7	95,4-96,0	95,3	95,0-95,5	98,3	98,2-98,5
Sorologia para HIV	98,6	98,4-98,7	96,9	96,7-97,1	98,0	97,8-98,1
Territorialização	59,0	58,3-59,8	69,6	69,1-70,2	79,5	79,0-79,9
Grupo	65,7	64,9-66,4	67,4	66,9-67,9	-	-
Busca ativa sintomas respiratórios	78,0	77,4-78,7	-	-	96,7	96,5-96,9
1ª amostra de escarro	-	-	57,0	56,4-57,5	70,8	70,3-71,3
Registro	75,1	74,5-75,8	94,7	94,4-95,1	83,7	83,3-84,0
Ficha de notificação	91,4	90,9-91,9	89,4	89,0-89,7	97,4	97,3-97,6
TDO	83,1	82,4-83,7	74,4	73,7-75,1	96,9	96,7-97,1
Busca ativa faltosos TDO	94,0	93,6-94,5	87,7	87,2-88,2	96,5	96,3-96,7
Processo de trabalho adequado	36,8	35,8-37,8	27,8	27,0-28,5	49,2	48,6-49,8

EPI: equipamentos de proteção individual; IC95%: intervalo de 95% de confiança; TDO: tratamento diretamente observado.

Sobre a prevalência de processo de trabalho adequado para diagnóstico, tratamento e monitoramento da TB, verifica-se que a disponibilidade de exames de baciloscopia, radiografia e sorologia do HIV manteve-se alta ao longo de todo o processo de avaliação, porém apresentando pequenas variações no ciclo II para raio-X, e nos ciclos II e III para sorologia do HIV. A busca ativa para os sintomas respiratórios aumentou de 78%, no ciclo I, para 96,7%, no ciclo III, e as UBS que coletavam a primeira amostra de escarro da TB também apresentaram aumento no percentual, de 57%, no ciclo II, para 70,8%, no ciclo III. A prevalência de processo de trabalho adequado teve um aumento de 12,4p.p., passando de 36,8%, no ciclo I, para 49,2%, no ciclo III (Tabela 2).

Conforme descrito na Tabela 3, todas as características estudadas apresentaram tendência crescente de infraestrutura adequada nos três ciclos do PMAQ-AB. Entre as regiões, a Sul (11,4p.p.; p < 0,001) e a Norte (9,0p.p.; p < 0,001) foram as que apresentaram maior aumento. Entre as categorias de IDH-M, a categoria mais baixa (abaixo de 0,554) demonstrou a menor tendência crescente (6,5p.p.; p < 0,001) e a alta (0,700-0,799) a maior tendência entre as categorias (8,5p.p.; p < 0,001). Chama a atenção que o maior aumento de infraestrutura adequada ocorreu nos municípios com cobertura de ESF de 50 a 75%, com crescimento anual de 8,5p.p. (p < 0,001), ao passo que os com cobertura de 100% atingiram 7,2p.p. ao ano (p < 0,001).

**Tabela 3:** Prevalência de unidades básicas de saúde (UBS) com infraestrutura adequada para diagnóstico e tratamento da tuberculose segundo características do município. Programa Nacional de Melhoria do Acesso e da Qualidade da Atenção Básica (PMAQ-AB), Brasil, 2012 (n = 13.842), 2014 (n = 24.055) e 2018 (n =28.939).

Características	Ciclo I (%)	Ciclo II (%)	Ciclo III (%)	Variação anual (p.p.)	Tendência	Valor de p
Brasil	12,0	29,8	54,6	7,0	Crescente	< 0,001
Região						
Norte	6,8	38,0	61,7	9,0	Crescente	< 0,001
Nordeste	3,2	26,8	51,0	8,3	Crescente	< 0,001
Sudeste	26,0	45,0	48,4	4,1	Crescente	< 0,001
Sul	7,8	24,0	76,5	11,4	Crescente	< 0,001
Centro-oeste	5,2	31,9	43,0	6,8	Crescente	< 0,001
Tamanho da população (habitantes)						
Até 10.000	11,0	31,7	49,2	6,3	Crescente	< 0,001
De 10.001 a 30.000	5,7	26,4	49,6	7,5	Crescente	< 0,001
De 30.001 a 100.000	8,5	29,0	55,0	7,8	Crescente	< 0,001
De 100.001 a 300.000	17,0	33,0	63,4	7,8	Crescente	< 0,001
Mais de 300.000	29,5	34,5	63,9	6,1	Crescente	< 0,001
IDH-M						
Baixo (até 0,554)	2,6	20,7	42,0	6,5	Crescente	< 0,001
Médio (de 0,555 a 0,699)	4,2	27,5	47,6	7,3	Crescente	< 0,001
Alto (de 0,700 a 0,799)	12,4	32,9	63,5	8,5	Crescente	< 0,001
Muito alto (0,800 ou mais)	26,7	40,0	76,7	8,3	Crescente	< 0,001
Cobertura de ESF (%)						
Até 50	26,3	39,0	62,7	6,0	Crescente	< 0,001
De 50,1 a 75	9,7	28,9	60,6	8,5	Crescente	< 0,001
De 75,1 a 99,9	8,1	30,9	56,4	8,0	Crescente	< 0,001
100	6,3	26,7	50,5	7,2	Crescente	< 0,001

ESF: Estratégia Saúde da Família; IDH-M: Índice de Desenvolvimento Humano Municipal; p.p.: pontos percentuais.

No que se refere ao processo de trabalho adequado (Tabela 4), todas as características estudadas apresentaram tendência crescente nos três ciclos do PMAQ-AB, com exceção da Região Centro-oeste, que permaneceu estável. Entre as demais regiões, a Norte (5,7p.p.; p < 0,001) apresentou maior aumento, seguida das regiões Sul (3,8p.p.; p < 0,001), Nordeste (3,4p.p.; p < 0,001) e Sudeste (3,1p.p.; p < 0,001). Tratando do tamanho populacional, os processos de trabalho demonstraram melhora crescente nos locais com até 300.000 habitantes, com crescimento observado entre 2,6 e 4,2p.p.; já os locais com população acima de 300.000 atingiram o aumento de 3,5p.p. ao ano, mas demonstraram oscilações no período. Entre as categorias de IDH-M, a categoria média (0,.555-0,699) demonstrou a menor tendência de crescimento (3,3p.p.; p < 0,001) e a alta (0,700-0,799) a maior tendência entre as categorias (4,1p.p.; p < 0,001). Por fim, observa-se uma heterogeneidade entre as categorias de cobertura de ESF. A maior tendência de processo de trabalho adequado ocorreu nos municípios com cobertura de 50,1 a 75% (4,1p.p.; p < 0,001) e o menor aumento ocorreu nos locais com cobertura inferior a 50% (2,4p.p.; p < 0,001).

**Tabela 4:** Prevalência de unidades básicas de saúde (UBS) com processo de trabalho adequado para diagnóstico e tratamento da tuberculose segundo características do município. Programa Nacional de Melhoria do Acesso e da Qualidade da Atenção Básica (PMAQ-AB), Brasil, 2012 (n = 13.842), 2014 (n = 24.055) e 2018 (n = 28.939).

Características	Ciclo I (%)	Ciclo II (%)	Ciclo III (%)	Variação anual (p.p.)	Tendência	Valor de p
Brasil	36,8	27,8	49,2	3,1	Crescente	< 0,001
Região						
Norte	28,0	18,7	50,8	5,7	Crescente	< 0,001
Nordeste	37,3	26,3	48,7	3,4	Crescente	< 0,001
Sudeste	39,3	33,4	53,5	3,1	Crescente	< 0,001
Sul	33,9	22,1	49,2	3,8	Crescente	< 0,001
Centro-oeste	34,5	28,4	34,6	0,52	Estável	0,056
Tamanho da população (habitantes)						
Até 10.000	32,9	26,1	44,7	2,6	Crescente	< 0,001
De 10.001 a 30.000	35,0	22,6	44,4	2,8	Crescente	< 0,001
De 30.001 a 100.000	33,0	24,1	47,8	3,8	Crescente	< 0,001
De 100.001 a 300.000	37,1	25,8	52,0	4,2	Crescente	< 0,001
Mais de 300.000	41,4	34,7	56,7	3,5	Crescente	< 0,001
IDH-M						
Baixo (até 0,554)	30,7	2,0	40,1	3,7	Crescente	< 0,001
Médio (de 0,555 a 0,699)	36,3	24,6	46,1	3,3	Crescente	< 0,001
Alto (de 0,700 a 0,799)	33,7	26,0	51,4	4,1	Crescente	< 0,001
Muito alto (0,800 ou mais)	40,3	41,6	61,0	3,6	Crescente	< 0,001
Cobertura de ESF (%)						
Até 50	42,0	32,6	52,0	2,4	Crescente	< 0,001
De 50,1 a 75	32,0	23,1	50,8	4,1	Crescente	< 0,001
De 75,1 a 99,9	33,6	26,4	49,2	3,5	Crescente	< 0,001
100	37,3	25,1	47,7	3,0	Crescente	< 0,001

ESF: Estratégia Saúde da Família; IDH-M: Índice de Desenvolvimento Humano Municipal; p.p.: pontos percentuais.

## Discussão

Ao longo dos três ciclos do PMAQ-AB, no período de 2012 a 2018, observa-se uma melhora gradual na proporção de infraestrutura e processo de trabalho adequado na APS para o diagnóstico, monitoramento e tratamento da TB. Entretanto, nenhum local está integralmente adequado. A maior tendência de infraestrutura adequada foi verificada na Região Sul, sendo que, no ano de 2018, 76,5% das UBS apresentavam a totalidade de instrumentos para o cuidado à TB. Em relação ao processo de trabalho adequado, a maior tendência foi na Região Norte e no ano de 2018, com 50,8% das equipes apresentando a totalidade de itens para o cuidado à TB.

A melhoria na incidência e mortalidade da TB, no Brasil durante o período de 2013 a 2018, está atribuída, em parte, às melhorias na infraestrutura e no processo de trabalho da APS no que se refere ao atendimento à doença ^15^^,^^16^^,^^17^. Tais melhorias foram alcançadas por meio de políticas públicas implementadas pelo PNCT, que visavam o fortalecimento da rede de diagnóstico e tratamento, a capacitação de profissionais de saúde, a mobilização social e a busca ativa de casos ^15^.

A melhoria da infraestrutura e do processo de trabalho no atendimento à TB pode contribuir para um controle mais efetivo da transmissibilidade da doença, resultando em uma redução da morbimortalidade da população afetada e na promoção da saúde pública ^15^. Uma infraestrutura adequada, com os equipamentos e suprimentos necessários, pode permitir o diagnóstico precoce e preciso da TB, além de facilitar a realização de exames complementares e o fornecimento de medicamentos aos pacientes. Já a melhoria do processo de trabalho, com a capacitação de profissionais de saúde e a implementação de estratégias de busca ativa de casos, pode contribuir para aumentar a sensibilização e o engajamento da população no controle da doença ^12^^,^^15^^,^^18^.

Contudo, é possível visualizar importantes marcadores de desigualdade no atendimento à TB, uma vez que somente 4 em cada 10 municípios com IDH-M baixo dispõem de infraestrutura e processo de trabalho adequado, enquanto entre aqueles com IDH-M alto, 6 em cada 10 têm infraestrutura adequada e praticamente 8 em cada 10 apresentam processo de trabalho adequado. Esses achados denotam preocupação, visto que as vulnerabilidades socioeconômicas são fatores de risco reconhecidos para diversos desfechos negativos em saúde, incluindo a TB ^19^^,^^20^^,^^21^. Além disso, o IDH-M é um indicador que merece destaque, pois em localidades com melhores índices, tanto a incidência quanto a mortalidade de TB costumam ser menores ^21^^,^^22^.

Um dado interessante de ser analisado é que, embora as desigualdades regionais em saúde sejam expressivas e persistentes, com destaque para piores indicadores na Região Norte ^23^^,^^24^, os achados deste estudo demonstram que essa região apresentou consistente melhora nos anos analisados. No último ciclo do PMAQ-AB, o Norte apresentou a segunda maior prevalência de infraestrutura e processo de trabalho adequado ao tratamento de TB. Todavia, é necessário considerar que, do total de UBS avaliadas, um percentual baixo era localizado na Região Norte e que as melhorias observadas podem não refletir a totalidade da macrorregião. Além disso, o progresso observado em termos de infraestrutura e processo de trabalho adequado não dialoga com as condições de saúde da população, em virtude da taxa de incidência e de mortalidade por TB serem superiores nessa região, assim como apresentar a menor média de IDH-M e menor cobertura de APS ^21^, o que demonstra a necessidade de contínuo fomento a esses indicadores para garantir efeito nos marcadores.

Também foram encontradas iniquidades em relação ao porte populacional dos municípios. As maiores cidades apresentaram os melhores resultados em termos de processos de trabalho e infraestrutura adequada para o atendimento da TB. Por um lado, o dado apresentado é positivo, em virtude das localidades com maior população, em geral, apresentarem dificuldades para organizar os serviços de saúde ^25^. Em contrapartida, já é histórico no cenário brasileiro que o desenvolvimento urbano, o crescimento econômico e o acesso aos serviços de saúde nem sempre ocorram concomitantemente ^26^. Nesse sentido, torna-se necessário que a contribuição de recursos da APS seja direcionada para atender às necessidades das localidades com piores indicadores de saúde, com o intuito de minimizar as disparidades visualizadas.

Apesar do coeficiente de incidência da doença ter apresentado oscilações e um discreto aumento no período de 2012 a 2018 (a cada 100 mil habitantes, 35,9 desenvolveram TB em 2012, 34,7 em 2014 e 36,9 em 2018) ^17^, parte desse aumento pode ser efeito da implementação de estratégias de busca ativa de casos no território e da maior procura por serviços de saúde pela população com sintomas respiratórios, o que possibilitou a ampliação das notificações dos casos ^15^^,^^27^. Entretanto, o sucesso no tratamento ainda não apresentou mudanças expressivas; de 2012 a 2018, em média 75% completaram o tratamento e 11,9% o abandonaram, sendo que, em 2020, esses valores aumentaram para 68,4% e 12,9%, respectivamente ^10^. Dessa forma, o sistema de saúde ainda apresenta fragilidades a serem superadas para alcançar êxito no tratamento efetivo da TB.

Ainda que a infraestrutura e o processo de trabalho adequado ao atendimento da TB venham apresentando melhora ao longo do período investigado, um terço das UBS e metade das equipes de saúde não atingiram esse critério. Em um cenário de fragilização da APS e do SUS como um todo, o alcance das metas estabelecidas no Plano Nacional Pelo Fim da Tuberculose torna-se passível de insucesso ^27^^,^^28^^,^^29^. Esses aspectos são de suma importância, uma vez que a articulação entre os pontos de atenção e monitoramento de casos ainda é precária e a continuidade do cuidado permanece como o principal desafio do controle da TB no Brasil ^12^^,^^30^.

As melhorias supracitadas reforçam a potência da APS como porta de entrada aos serviços de saúde, em uma perspectiva de fomentar o acesso da população e ampliar a resolutividade das ações de acordo com as necessidades locais de cada território assistido ^31^^,^^32^. Além disso, observa-se que o PMAQ-AB é, de certa forma, o precursor desses avanços, uma vez que os indicadores estabelecidos sempre objetivam a melhora na qualidade da assistência e o estabelecimento de um padrão comparável em todo o país ^31^. Contudo, a partir do ano de 2017, houve alterações na política da APS e, por meio da instituição do programa Previne Brasil em 2019, ocorreu a extinção do PMAQ-AB, que criou novas formas de avaliação e novos parâmetros ^33^^,^^34^. Somadas a essas questões, as medidas de austeridade fiscal, como a *Emenda Constitucional nº 95*, dificultam ainda mais a adequação da assistência aos princípios e diretrizes do SUS, visto que os repasses financeiros não são suficientes para suprir a carência dos serviços em termos de equipamentos e profissionais para atender às demandas e às necessidades da população ^29^^,^^35^^,^^36^.

Paralelo a essas modificações na política de saúde, o cenário pandêmico também impactou negativamente na assistência prestada aos indivíduos acometidos pela TB ^37^^,^^38^ e, possivelmente, os avanços alcançados no período investigado para a infraestrutura e o processo de trabalho adequado ao cuidado da TB foram afetados e até mesmo descontinuados. Dados do Ministério da Saúde apontam um crescimento tanto no número de pessoas que abandonaram o tratamento quanto no número de óbitos em decorrência da TB ^5^^,^^10^. Durante esse período, os esforços dos serviços de saúde foram redirecionados para o atendimento a pacientes com COVID-19; dessa forma, os serviços essenciais de assistência ao paciente com TB foram restringidos, devido à redução de recursos financeiros, profissionais e insumos ^37^^,^^38^. Em vista desses aspectos, preocupa a maneira como o sistema de saúde lidará com os impactos da pandemia e reestruturará os serviços de diagnóstico e tratamento da TB, com vistas a alcançar as metas estabelecidas pelo PNCT.

Os pontos fortes deste estudo incluem o recorte temporal analisado com base em dados nacionais da APS e a importância da temática. Sobre as limitações, o desfecho considerou infraestrutura e processo de trabalho adequado ou inadequado com o objetivo de estabelecer um panorama geral; contudo, apresenta fragilidades quanto ao esclarecimento de maiores detalhes, pois sugere um desfecho sintético com poucas variáveis, o que facilita a compreensão e construção e reduz o detalhamento. Além disso, não foi possível analisar a relação da infraestrutura e do processo de trabalho adequado com o cuidado recebido pelos usuários com TB na APS, pois os dados sobre esses indivíduos não estão disponíveis no PMAQ-AB. Outro ponto a ser mencionado é que, no primeiro ciclo do PMAQ-AB, as UBS e equipes eram convidadas a participar e, nos ciclos II e III, todas as unidades e equipes deveriam participar; dessa forma, o ciclo I poderia ter viés de seleção, considerando que, como primeiro ciclo, talvez apenas as melhores unidades e equipes dos municípios tenham aderido a ele.

Com base nos resultados apresentados, é possível concluir que houve uma tendência significativa de melhora na infraestrutura e no processo de trabalho adequado na APS para o diagnóstico, monitoramento e tratamento da TB no Brasil. A criação do PNCT e a avaliação contínua do PMAQ-AB contribuíram para esses avanços observados. No entanto, ainda há muito a ser feito para que todas as UBS e equipes de saúde estejam completamente adequadas para o cuidado da doença, o que demonstra a necessidade de um esforço conjunto para compreender as múltiplas realidades dos territórios, com vistas a aprimorar as condições de saúde e reduzir as disparidades regionais existentes. Além disso, com o fim do PMAQ-AB e o novo modelo de financiamento da APS pelo Previne Brasil, surgem novos desafios para o aperfeiçoamento contínuo da qualidade do atendimento à TB. Nesse sentido, questiona-se como será o processo de avanço nessa área e como serão gerenciados os recursos para garantir a sustentabilidade das melhorias alcançadas. É fundamental que sejam criadas estratégias e políticas públicas que mantenham o compromisso com esse avanço do atendimento à TB na APS, a fim de garantir a eficácia das medidas de controle e prevenção da doença e, consequentemente, promover a saúde da população brasileira.
